# Evaluating the Role of Autochthonous Lactic Acid Bacteria and Ripening Conditions on Nitrosamine Inhibition in Sucuk Over Storage Time

**DOI:** 10.1002/fsn3.70851

**Published:** 2025-08-25

**Authors:** Selen Sallan, Zeynep Feyza Yılmaz Oral, Yağmur Akyol, Güzin Kaban, Mükerrem Kaya

**Affiliations:** ^1^ Department of Food Processing, Bandırma Vocational School Bandırma Onyedi Eylül University Balıkesir Türkiye; ^2^ Department of Food Engineering, Faculty of Agriculture Atatürk University Erzurum Türkiye

**Keywords:** fermented sausage, lactic acid bacteria, NDMA, nitrosamine, NPIP, ripening rate

## Abstract

The study determined the effects of lactic acid bacteria (LAB) (LS: *Latilactobacillus sakei* S15, LP: *Lactiplantibacillus plantarum* S91, PP: *Pediococcus pentosaeceus* S128b), ripening condition (slow or rapid ripening), and storage time (0, 30 or 90 days) on microbiological and physicochemical properties of sucuk, a kind of dry fermented sausage. In addition to these factors, the effect of cooking time (raw, 1 min or 3 min) on nitrosamine content was also investigated. LAB count showed a significant change during storage. LS and LP caused a greater decrease in pH than PP. In addition, LS and LP led to a higher a* value. Thiobarbituric acid reactive substances values of LS and LP groups were lower than those of control and PP groups. Rapid ripening caused lower residual nitrite. The starter culture did not show a significant effect on N‐nitrosopiperidine (NPIP), N‐nitrosopyrrolidine (NPYR), and N‐nitrosomethylethylamine (NMEA). In the presence of LS, LP, or PP, N‐nitrosodimethylamine (NDMA) decreased on the 30th day, and no significant change was observed on the 90th day. A significant decrease was detected in NDMA, NPYR, and NPIP as the storage period progressed. At the 0th day of storage, the NDMA level increased when cooked for 1 min, but an increase was observed only after 3 min of cooking in the samples stored for 90 days. In conclusion, the autochthonous strains showed good adaptation in sucuk and affected only NDMA.

## Graphical abstract

The autochthonous lactic acid bacteria inhibited NDMA. Differences were noted among strains in terms of acid formation. At the end of ripening, slow ripening yields higher residual nitrite than rapid ripening.

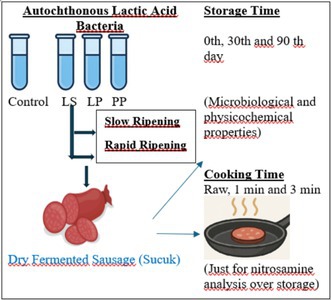

## 1 Introduction

Nitrosamine is a kind of hazardous chemical contaminant that can exert severe carcinogenic, teratogenic, and mutagenic effects on humans (Xie et al. 2023). N‐nitrosodiethylamine (NDEA) and N‐nitrosodimethylamine (NDMA) have been categorized as Group 2A (probably) carcinogenic to humans. N‐nitrozopiperidine (NPIP), N‐nitrosodibutylamine (NDBA), N‐nitrosopyrrolidine (NPYR), and N‐nitrosomorpholine (NMOR) are Group 2B (possibly) carcinogenic compounds for humans (IARC 2020). Group 2A indicates that there is strong evidence that it can cause cancer in humans, but at present it is not conclusive, while Group 2B means that there is some evidence that it can cause cancer in humans, but at present it is far from conclusive (IARC 2025).

Among meat products, fermented sausages have a special importance in terms of nitrosamines due to their production conditions and content. NDMA, NPIP, and NPYR are extensively determined in these products (Herrmann, Duedahl‐Olesen, and Granby 2015; Kaban, Polat, et al. 2022; Kızılkaya et al. 2023). Fermented sausages are generally divided according to two distinct ripening processes: rapid or slow (Demeyer 2007). Since nitrosamine formation is affected by residual nitrite and ingoing nitrite, the rate and level of acidification throughout fermentation are important for both nitrite degradation and nitrosamine formation in both product types. On the other hand, nitrosamine formation is significantly influenced by the secondary amines produced by proteolysis during ripening. In addition, the cooking method and cooking degree are another important issues that affect the nitrosamine level (Sallan et al. 2023; Xie et al. 2023). Although fermented sausages are, in general, consumed raw, sucuk, the main dry fermented sausage type widely consumed in Turkey, is cooked before consumption due to consumption patterns (Kızılkaya et al. 2023). Previous studies have shown that cooking causes significant increases in the nitrosamine levels in sucuk (Sallan et al. 2019, 2023; Kızılkaya et al. 2023). These factors make it necessary to continue developing strategies to prevent nitrosamine from forming in this product.

To inhibit the nitrosamine formation, a great deal of research has been conducted on the addition of antioxidant‐rich substances including polyphenols, ascorbic acid, erythrocytic acid, and tocopherol to the composition of fermented sausages (Herrmann, Granby, and Duedahl‐Olesen 2015; Wang et al. 2015; Sallan et al. 2019, 2020). In a study examining the effects of epigallocatechin gallate, gallic acid, and epigallocatechin on NDMA formation, it was determined that these compounds had an inhibitory effect, with epigallocatechin, gallic acid, and epigallocatechin gallate having the highest effect, respectively (Deng et al. 2019). Similarly, several investigations indicate that ascorbic acid reduces the development of nitrosamines (Pourazrang et al. 2002; Li et al. 2013); yet, studies also suggest that ascorbic acid might act as a pro‐oxidant and catalyze the formation of nitrosamines (Yen et al. 2002; Combet et al. 2010). Studies were also carried out to reduce the formation of nitrosamine by non‐thermal technologies such as ionize radiation, which suggests that these techniques can lead to the production of unwanted flavors and odors and certain toxic compounds in the final product (Jo et al. 2003; Rabie and Toliba 2013). In recent years, some autochthonous lactic acid bacteria (LAB) strains have been shown to directly or indirectly reduce or prevent the formation of nitrosamine (Kim, Kang, et al. 2017; Kim, Kim, et al. 2017; Sun et al. 2017; Xiao et al. 2018; Sallan et al. 2020; Shao et al. 2021).

In the current study, it was aimed to reveal the effects of different ripening conditions (slow or rapid ripening), storage time (0, 30, and 90 days) and autochthonous LAB strains (*Latilactobacillus sakei* S15 (LS), *Lactiplantibacillus plantarum* S91 (LP), *Pediococcus pentosaeceus* S128b (PP)) on nitrosamine content, microbiological (lactic acid bacteria and *Micrococcus/Staphylococcus*) and physicochemical properties (pH, a_w_, residual nitrite, TBARS and color) of sucuk. The aim of this study was also to determine the effect of cooking degree on the nitrosamine content of sucuk, in addition to LAB strains, ripening rate, and storage time.

## 2 Materials and Methods

### 2.1 Material

Meat and fat obtained from the round parts of cow carcasses were used as raw material. After excess fat and other connective tissues were removed, the meat and fat were chopped into small pieces. The prepared small pieces of the meat were mixed by hand in a stainless steel vessel, then vacuum packaged and stored at −20°C. Salt, garlic, and spices were also obtained from Erzurum market. NaNO_2_ was used as a curing agent in the sucuk production.

*Latilactobacillus sakei* S15 (Accession No: KR025387), *L. plantarum* S91 (Accession No: KT327838), *P. pentosaeceus* S128b (Accession No: KT327865) (Kaya et al. 2017), which were isolated from sucuk before and genetically identified by 16S rRNA gene sequencing, were used as starter culture. Inoculation was performed at a minimum of 10^6^ cfu/g. In the nitrosamine analyses, the internal standard was Nitrosodipropylamine‐d14 (N525482; TRC, North York, Canada) and a nitrosamine mix was obtained from EPA 521 Supelco, Bellefonte PA.

### 2.2 Sucuk Production

The sucuk batters were based on the formulation provided by Sallan et al. (2023) for kg of beef meat and beef fat (80:20) and included 20 g salt, 4 g sucrose, 10 g garlic, 9 g cumin, 2.5 g allspice, 7 g paprika, 5 g black pepper, and 0.15 g sodium nitrite. The study was carried out with 3 replications, based on three different autochthonous lactic acid bacteria (LS: *L. sakei* S15, LP: *L. plantarum* S91, PP: *P. pentosaeceus* S128b), two different ripening rates (slow and rapid ripening), storage time (0, 30 or 90 days) and cooking time (0, 1 or 3 min) factors at three different times.

Starter culture was not used in the first group of sucuk batter and was evaluated as the control. LS, LP, and PP were inoculated into the second, third, and fourth sucuk batters, respectively. Thus, in total, of 24 batters, 12 pieces of sucuk batters (4 treatment (control, LP, LS, PP) × 3 replications (3 different times)) were prepared for both slow ripening and rapid ripening.

Sucuk batters (5 kg per group) were prepared in a laboratory type cutter (MADO Typ MTK 662, Dornhan/Schwarzwald) at slow speed and filled into collagen casings (38 mm; Naturin GmbH and Co., Weinheim Germany) weighing 200 ± 10 g using a laboratory type filling machine (MADO Typ MTK 591, Dornhan/Schwarzwald). After 4 h of equilibration, the prepared sucuk samples were subjected to ripening in a climate unit (REICH Thermoprozesstechnik GmbH, Schechingen, Germany) that can be automatically controlled of temperature, air flow and relative humidity.

In rapid ripening, the initial fermentation temperature was set at 24°C ± 1°C and the relative humidity at 90% ± 2% for the first day of ripening. On the second day, only the temperature was reduced to 22°C ± 1°C. The temperature was set to 20°C ± 1°C and relative humidity to 88% ± 2% on the third day of ripening, whereas, on the fourth day, the temperature and relative humidity were set to 18°C ± 1°C and 86% ± 2%, respectively. In the last 3 days of ripening, the temperature was held at 18°C ± 1°C and the relative humidity was adjusted to 84% ± 2%, 80% ± 2%, and 80% ± 2%, respectively. According to this program, the total ripening period lasted 7 days.

In slow ripening, the initial fermentation temperature was set at 18°C ± 1°C and the relative humidity was set at 90% ± 2% on the first day of ripening, and the fermentation temperature and relative humidity were not changed for 4 days. On the fifth and sixth days of fermentation, the temperature was adjusted to 16°C and relative humidity to 88% ± 2%. On the seventh day of fermentation, only the relative humidity was lowered to 86% ± 2%, and on the eighth and ninth days, the temperature was held constant at 16°C ± 1°C and relative humidity at 86% ± 2%. According to this program, the total ripening period lasted 9 days. The sucuk samples obtained at the end of ripening were subjected to vacuum‐packaging (Multivac A300/16, Sepp Haggenmüller, Germany) with polyamide/polyethylene material (Sudpack Verpackungen GmbH Co, Germany), then stored at 4°C.

### 2.3 Cooking Procedure and Sampling

The samples were sliced into 0.5 mm thickness at the end of production. The cooking process was conducted on a hot plate. Prior to cooking, the surface temperature of the hot plate was increased to 180°C, and the temperature was monitored using a digital thermocouple (Testo 926, Testo, Titisee‐Neustadt, Germany). The samples were subjected to three different cooking levels (raw, 1 or 3 min) on certain days of storage (0, 30, and 90 days) for nitrosamine analysis. The cooking time was set to 1 or 3 min, with 0.5 or 1.5 min for each surface. Samples that were not subjected to cooking (raw) were considered the control group. Following the homogenizing of each sample, they were transferred to a glass jar and stored at 20°C until analyzed.

### 2.4 Analyses

Samples taken from the final product (Day 0 of storage) and at storage periods (30 and 90 days) were analyzed for color, water activity, pH, residual nitrite, and thiobarbituric acid reactive substance (TBARS). Nitrosamine analysis was applied to both raw and cooked samples at all storage periods.

#### 2.4.1 Lactic Acid Bacteria, *Micrococcus/Staphylococcus*, and Enterobacteriaceae Counts

MRS (De Man Rogosa Sharpe Agar) agar medium was utilized for lactic acid bacteria count of the samples. After inoculation of MRS plates with appropriate dilutions by spread method, the plates were incubated at 30°C for 2 days under anaerobic conditions (Anaerocult A). After incubation, lactic acid bacteria count was detected by considering the number of catalase (−) colonies (Baumgart et al. 1993). MSA (Mannitol Salt Red) agar medium was used for *Micrococcus/Staphylococcus* count. The plates were incubated aerobically for 2 days at 30°C. After incubation, *Micrococcus/Staphylococcus* count was determined by taking catalase (+) cocci into consideration (Baumgart et al. 1993). VRBD (Violet Red Bile Dextrose) Agar was used for Enterobacteriaceae count. VRBD agar plates were inoculated by spread method and under anaerobic conditions, petri plates were incubated for 2 days at 30°C (Anaerocoult A) (Baumgart et al. 1993).

#### 2.4.2 pH

After weighing 10 g of the analyzed sample, 100 mL of pure water was added and the mixture was homogenized with ultra‐turrax. The pH value of the homogenate was determined using a pH meter. The pH meter was calibrated with buffer solutions (pH: 4.0 and 7.0) prior to use (Sallan 2023).

#### 2.4.3 Water Activity (a_w_)

Water activity device (Novasina AG CH‐8853, Switzerland) was used to detect the a_w_ values of the samples. The device was calibrated at 25°C with 6 different salt solutions before use. a_w_ was measured at 25°C after the samples were placed in specific plastic containers and placed in the device's measuring cabinet.

#### 2.4.4 Color (L*, a*, and b*)

L*, a*, and b* values of the samples were observed via a colorimeter (Minolta Co., Osaka, Japan) with *C D65 illuminant, an aperture size of 8 mm, and a standard observed of 2°. The samples were sliced into 1 cm thickness before measurement. The measurements were carried out following a 30 min blooming time.

#### 2.4.5 Thiobarbituric Acid Reactive Substances (TBARS)

The homogenized samples were subjected to the method described by Kilic and Richards (2003). The findings as μmol MDA/kg were obtained.

#### 2.4.6 Residual Nitrite Analysis

A 200 mL flask was filled with a 10 g sample and 50 mL of ultrapure water (50°C–60°C) to measure the residual nitrite. Following a further 15 min of stirring, 50 mL of acetonitrile was added, then the volume was adjusted to 200 mL with ultrapure water. After being filtered through a 0.45 m filter using nitrite‐free/nitrate‐free filter paper (MN 640 de, Macherey‐Nagel), the samples were transferred into vials. A diode array detector (DAD) (Agilent Technology, Santa Clara, CA, USA) in conjunction with high‐performance liquid chromatography (HPLC) was utilized for the measurement. The system's flow rate was 2 mL/min. The wavelength of UV was 220 nm. The injection volume was determined to be 100 L. Based on the nitrite standard calibration, the results are explained in mg/kg (NMKL 2000).

#### 2.4.7 Nitrosamine Analysis

The homogenized sample was weighed into centrifuge tubes and homogenized after adding 0.1 M NaOH solution. After that, methanol was added and the homogenate was centrifuged at 10,000 rpm at 4°C. After concentrating the sample, the extract was filtered using glass microfibre (70 mm diameter, Whatman GF Healthcare Life Sciences, UK), and then was transferred to a ChemElut column (Agilent ChemElut, 20 mL, Unbuffered, USA) by adding a 20% NaCl solution. Dichloromethane (50 mL) was utilized in order to elute the column after a 20‐min equilibration period. Kuderna Danish equipment was used to concentrate the eluent to 1 mL. Nitrosamines were detected by GC–MS after the concentrate was evaporated at 40°C using a nitrogen evaporator (N‐EVAPTTM 111, Clarion Safety Systems).

In the system, carrier gas was helium and DB‐5MS (30 m × 0.25 mm × 0.25 μm, Agilent Tech) was the column and operated with SIM mode. As an internal standard, N‐nitrosodipropylamine‐d14 was employed. After holding the oven temperature at 50°C for 2 min, it increased to 100°C at a rate of 3°C per min and remained there for 5 min before rising to 250°C at a rate of 20°C per min. Nitrosamine mix (EPA 521 Nitrosamine Mix, Supelco, Bellefonte, PA, USA) was used for identification and nitrosamine levels (NPIP, NDPA, NDEA, NMEA, NDBA, NPYR, NDMA) were determined at ppb level (Wang et al. 2015). Limit of detection (LOD), linear range, correlation coefficient (*R* ^2^), limit of quantification (LOQ), recovery (%), linear equality, and relative standard deviation (RSD %) of the nitrosamines are illustrated in Table S1.

#### 2.4.8 Statistical Analysis

In the study, autochthonous LAB (control, LP, LS and PP), ripening rate (slow or rapid ripening) and storage time (0, 30, and 90 days) were taken as factors, and the experiments were carried out at three different times in a 4 × 2 × 3 factorial design with 3 replicates (3 blocks) according to a randomized complete blocks experimental plan. In addition to these factors, cooking time (raw, 1‐ or 3‐min) was included as a factor just for nitrosamine analysis. Starter culture addition, ripening rate, storage time, and cooking time were assessed as independent variables. A two‐way analysis of variance (ANOVA) using a general linear model was applied to the results, treating the replications as a random effect and the factors and their interactions as main effects. The statistical program SPSS version 24 (SPSS Inc., Chicago, II, USA) was used to compare the means of the main sources of variation and the interactions that were determined to be significant using the Duncan multiple comparison test (at *p* < 0.05 level).

## 3 Results and Discussion

### 3.1 Lactic Acid Bacteria, *Micrococcus/Staphylococcus*, and Enterobacteriaceae

**FIGURE 1:**
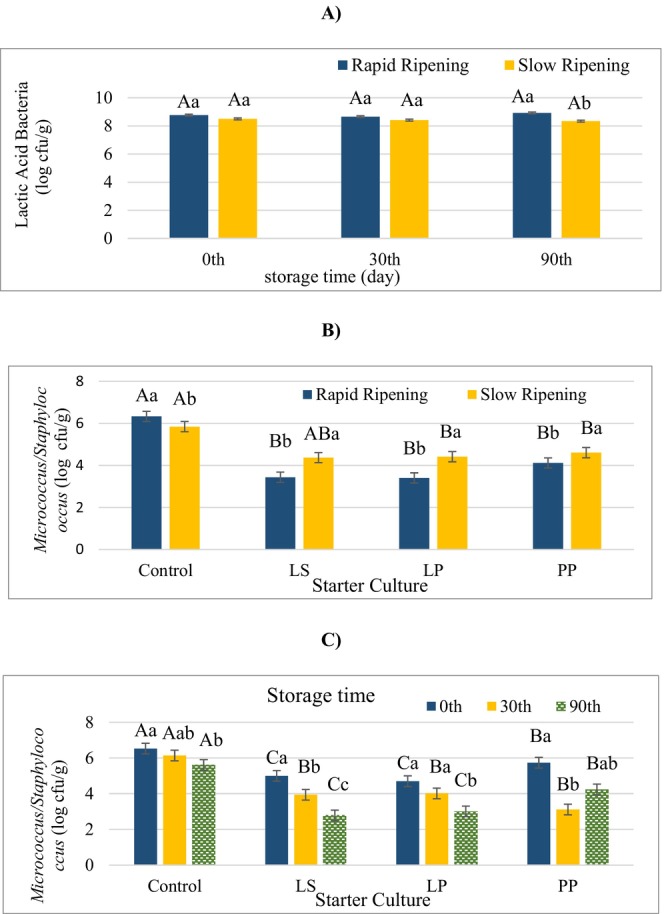
The effects of the interactions of ripening rate × storage time on the lactic acid bacteria counts (A), ripening rate × starter culture on the *Micrococcus/Staphylococcus* counts (B), and storage time × starter culture on the *Micrococcus/Staphylococcus* counts (C). (A) a and b: Different lowercase letters indicate significant differences between samples produced in the different ripening rate for the storage time. A–B: Different capital case letters indicate significant differences between samples during the storage time for different ripening rates; (B) a and b: Different lowercase letters indicate significant differences between samples produced in the different ripening rate for starter culture, A–B: Different capital letters indicate significant differences between samples produced with different starter culture for ripening rate; (C) a–c: Different lowercase letters indicate significant differences between samples during storage time for starter culture, A–C: Different capital letters indicate significant differences between samples produced with different starter culture for storage time (*p* < 0.05).

Regarding lactic acid bacteria (LAB), the control group had a lower mean than any of the other groups (LS, LP, PP) (Table 1). On the other hand, as can be seen in Figure 1A, while the ripening rate on Days 0 and 30 of storage had no significant influence on the lactic acid bacteria count, slow ripening resulted in a lower LAB mean value than rapid ripening on Day 90. However, the mean LAB number was not below 8 log cfu/g in both groups. These results show that spontaneous LAB in the control group also showed good development. However, the rate and degree of acid formation by LAB during fermentation are of great importance in terms of product safety, and this stage plays a critical role in the inhibition of pathogenic bacteria. Therefore, it is important to use selected strains as starter cultures for controlled fermentation. These microorganisms are important in terms of ensuring product safety and the development of sensory properties in dry fermented sausages such as sucuk, salami, and rohwurst (Agüero et al. 2020; Petrović et al. 2025). *Micrococcus/Staphylococcus* (M/S) is another group of microorganisms that are technologically significant in fermented sausages. In this study, the interaction of ripening rate × starter culture was found to be significant on M/S count of sucuk (Table 1). The control group gave higher values in both slow and fast ripening, and there was no significant difference between the control and LS in slow ripening (Figure 1B). Since M/S are acid‐sensitive microorganisms, these results are assumed to be due to pH differences (Kaban, Sallan, et al. 2022; Akköse et al. 2023). On the other hand, the control group gave higher M/S values during storage than the other groups (LS, LP, PP). The number of M/S generally decreased as the storage period progressed in all treatment groups (Figure 1C).

**TABLE 1:** Overall effects of ripening rate, starter culture and storage time on LAB, M/S, pH, a_w_, TBARS, residual nitrite, L*, a*, and b* of sucuk (mean ± SE).

Treatment	*n*	LAB (log cfu/g)	M/S (log cfu/g)	pH	a_w_	TBARS (μmol MDA/kg)	Residual nitrite (mg/kg)	L*	a*	b*
Ripening rate (RR)										
Rapid	36	8.78 ± 0.04a	4.33 ± 0.12b	4.86 ± 0.01b	0.902 ± 0.001a	11.881 ± 0.23a	8.400 ± 0.21b	38.57 ± 0.34a	11.37 ± 0.15a	8.78 ± 0.102b
Slow	36	8.42 ± 0.04b	4.81 ± 0.12a	5.05 ± 0.01a	0.894 ± 0.001b	10.645 ± 0.23b	10.149 ± 0.21a	37.97 ± 0.34a	11.04 ± 0.15a	9.60 ± 0.102a
Significance		**	**	**	**	**	**	ns	ns	**
Starter culture (SC)										
Control	18	8.25 ± 0.06b	6.09 ± 0.17a	5.22 ± 0.02a	0.898 ± 0.001b	11.89 ± 0.33a	10.02 ± 0.30a	36.55 ± 0.48b	10.49 ± 0.22b	8.85 ± 0.144b
LS	18	8.76 ± 0.06a	3.91 ± 0.17b	4.77 ± 0.02c	0.895 ± 0.001c	10.63 ± 0.33b	8.21 ± 0.30b	39.41 ± 0.48a	11.72 ± 0.22a	9.62 ± 0.144a
LP	18	8.74 ± 0.06a	3.91 ± 0.17b	4.77 ± 0.02c	0.897 ± 0.001bc	10.72 ± 0.33b	9.49 ± 0.30a	39.43 ± 0.48a	11.66 ± 0.22a	9.40 ± 0.144a
PP	18	8.65 ± 0.06a	4.37 ± 0.17b	5.06 ± 0.02b	0.902 ± 0.001a	11.81 ± 0.33a	9.38 ± 0.30a	37.69 ± 0.48b	10.95 ± 0.22b	8.89 ± 0.144b
Significance		**	**	**	**	**	**	**	**	**
Storage time (day) (ST)										
0	24	8.63 ± 0.05a	5.491 ± 0.15a	4.97 ± 0.01a	0.896 ± 0.001b	8.49 ± 0.29c	12.25 ± 0.26a	38.45 ± 0.41ab	11.83 ± 0.19a	8.76 ± 0.125b
30	24	8.54 ± 0.05a	4.31 ± 0.15b	4.94 ± 0.01a	0.899 ± 0.001a	11.60 ± 0.29b	9.82 ± 0.26b	37.40 ± 0.41b	11.44 ± 0.19a	8.87 ± 0.125b
90	24	8.63 ± 0.05a	3.91 ± 0.15b	4.97 ± 0.01a	0.900 ± 0.001a	13.69 ± 0.29a	5.76 ± 0.26c	38.96 ± 0.41a	10.36 ± 0.19b	9.45 ± 0.125a
Significance		ns	**	ns	**	**	**	*	**	**
RR × SC		ns	*	**	**	ns	*	ns	ns	ns
RR × ST		*	ns	ns	*	**	**	ns	ns	**
SC × ST		ns	*	ns	ns	ns	ns	ns	ns	ns

*Note:* a–b: means marked with different letters in the same column are statistically different for each factor (ripening rate, starter culture and storage time) (*p* < 0.05).

Abbreviations: LAB, lactic acid bacteria; LP, *Lactiplantibacillus plantarum* S91; LS, *Latilactobacillus sakei* S15; M/S, *Micrococcus*/*Staphylococcus*; ns, not significant; PP, *Pediococcus pentosaeceus* S128b; SE, standard error.

* *p* < 0.05.

** *p* < 0.01.

In every group, the number of Enterobacteriaceae was less than the detectable limit (< 2 log cfu/g) during the storage time (0, 30, and 90 days) of sucuk samples produced at different ripening rates (slow and rapid ripening) using autochthonous strains (control, LP, LS, PP) (data not shown). The pH decrease in fermented sausages is a crucial factor for the inhibition of members of this family (Wang et al. 2021; Yılmaz Oral and Kaban 2021; Akköse et al. 2023). In this study, both spontaneous LAB in the control group and autochthonous strains adapted well to the fermentation environment and showed good growth. Thus, they made an important contribution to product safety.

### 3.2 pH and a_w_

**FIGURE 2:**
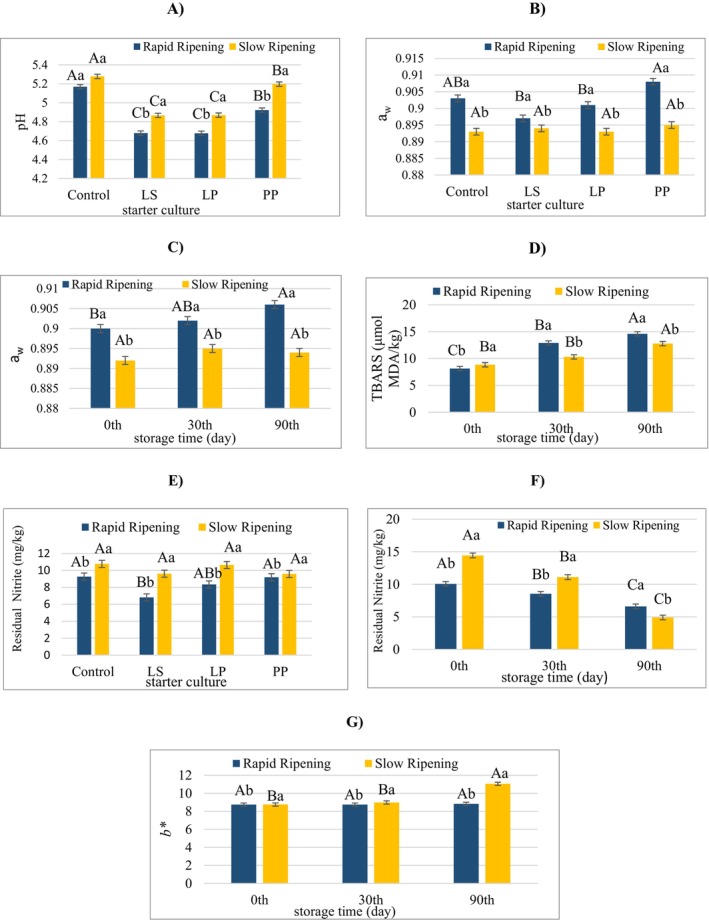
The effects of the interactions of the factors (ripening rate, storage time, or starter culture) on pH (A), a_w_ (B, C), TBARS (D), residual nitrite (E, F), or b* value (G). a and b: Different lower case letters indicate significant differences between samples produced at different ripening rates for starter culture. A–C: Different capital letters indicate significant differences between samples produced with different starter cultures for the ripening rate (for A, B, and E). a and b: Different lower case letters indicate significant differences between samples produced at the different ripening rates for the storage time. A and B: Different capital case letters indicate significant differences between samples during the storage time for the ripening rates (for C, D, F, and G) (*p* < 0.05).

A significant hurdle effect in fermented sausages is pH. In the study, the storage time had no significant influence in terms of the pH (*p* > 0.05). However, both the use of the starter culture and the ripening rate had a significant influence on the pH (*p* < 0.05) (Table 1). While the ripening rate had no important effect on pH in the control group, rapid ripening in the starter culture groups resulted in lower pH values. Among the strains, the pH decrease was smaller when PP was used in both ripening conditions, compared to LS and LP (Figure 2A). In contrast, the storage time had no important impact on the pH (*p* > 0.05) (Table 1). Another important parameter in fermented sausages is a_w_. In sucuk groups with and without starter culture, slow ripening showed lower a_w_ values compared to rapid ripening. Nevertheless, there was no significant change in the groups regarding a_w_ in slow ripening. In fast ripening, only PP gave higher a_w_ values compared to LP and LS (Figure 2B). Slow ripening also did not affect the a_w_ during storage (Figure 2C).

### 3.3 Thiobarbituric Acid Reactive Substances (TBARS) and Residual Nitrite

Starter culture factor had a significant effect on TBARS value of sucuk (*p* < 0.01). The TBARS values of LS and LP groups were lower than those of control and PP groups (Table 1). Similarly, Akköse et al. (2023) reported that mixed culture containing *L. plantarum* GM77 reduced the TBARS value of sucuk. In another study conducted on sucuk, it was determined that the mixed culture containing *L. sakei* S15 decreased the TBARS value in the study by Kaban, Sallan, et al. (2022). As can be seen in Figure 2D, at the beginning of storage, slow ripening gave higher TBARS values, while on other days of storage, it showed lower mean TBARS values compared to rapid ripening. Akköse et al. (2023) also observed that slow ripening gave higher TBARS values. In addition, in our study, in rapid ripening, TBARS values increased as time proceeded, but in slow ripening, an increase was obtained only on the 90th day (Figure 2D). The changes in pH and residual nitrite levels over the storage period are believed to be the cause of this finding.

Ripening rate had a significant influence on residual nitrite (*p* < 0.05). Rapid ripening resulted in lower mean residual nitrite values compared to slow ripening (*p* < 0.05) (Table 1). However, in rapid ripening, the lowest residual nitrite mean was determined in the LS group, yet this value did not differ from the mean residual nitrite value of LP. The use of starter culture in slow ripening had no significant effect on residual nitrite, whereas, in rapid ripening, the lowest residual nitrite level was detected in the presence of LS. However, the mean value of LS was not statistically different from the LP nitrite level (Figure 2E). It is thought that the differences in nitrite levels between the groups are due to pH differences. Nitrous acid concentration rises with decreasing sausage pH, causing a corresponding change in nitrite concentration depending on the pH level (Honikel 2010; Sallan et al. 2023). Therefore, pH decrease during fermentation is of great importance. Previous investigations demonstrated that the rapid pH decrease may bring about a faster nitrite depletion during fermentation (Wang et al. 2013; Chen et al. 2016). In the current study, as can be seen in Figure 2F, both slow and rapid ripening showed a decrease in residual nitrite as the storage time progressed. Slow ripening had a higher mean residual nitrite value at the beginning of the storage and on Day 30, while the opposite was observed at the end of the storage time (Figure 2F). Aside from the effect of pH, nitrite depletion was reported to be associated with initial nitrite levels, raw meat type, processing conditions, storage, and the presence of reducing agents (Wang et al. 2022).

### 3.4 Color (L*, a*, and b*)

Starter culture had a very significant impact on L* value, which is a marker of brightness (*p* < 0.01). There was no significant difference in L* value between the sucuk samples belonging to the control group and PP group. However, LS and LP gave higher mean values (Table 1). Likewise, Akköse et al. (2023) reported that *L. plantarum* increased the L* value of sucuk at the end of ripening. According to a study on sucuk, the control group had lower L* values than the groups with starter culture (Kaban, Sallan, et al. 2022). The L* value was not significantly affected by the ripening rate (*p* > 0.05), but some differences in L* were observed during storage. On the other hand, a* value, indicating redness in sucuk, was not influenced by ripening rate; however, it significantly decreased on the 90th day of storage (Table 1). It is estimated that this result is due to the partial oxidation of nitrosomyoglobin, which is the cured meat color, during storage (Honikel 2008). Among the starter culture groups, LS and LP autochthonous strains gave higher a* values (*p* < 0.05). This result is thought to be due to the lower pH of LS and LP groups compared to the others (control and PP) (Table 1) and the acceleration of nitric oxide (NO) formation, which plays a role in the formation of nitrosomyoglobin (Honikel 2008). Differences were also observed between the starter culture groups in terms of b* values, which is an indicator of yellowness. It was determined that b* value of groups with LS and LP was higher than the other groups (control and PP) (Table 1). In slow ripening, the highest value was seen on the 90th day, whereas in rapid ripening, b* value remained unchanged throughout the storage (Figure 2G).

### 3.5 Nitrosamine

#### 3.5.1 N‐Nitrosodimethylamine (NDMA)

**FIGURE 3:**
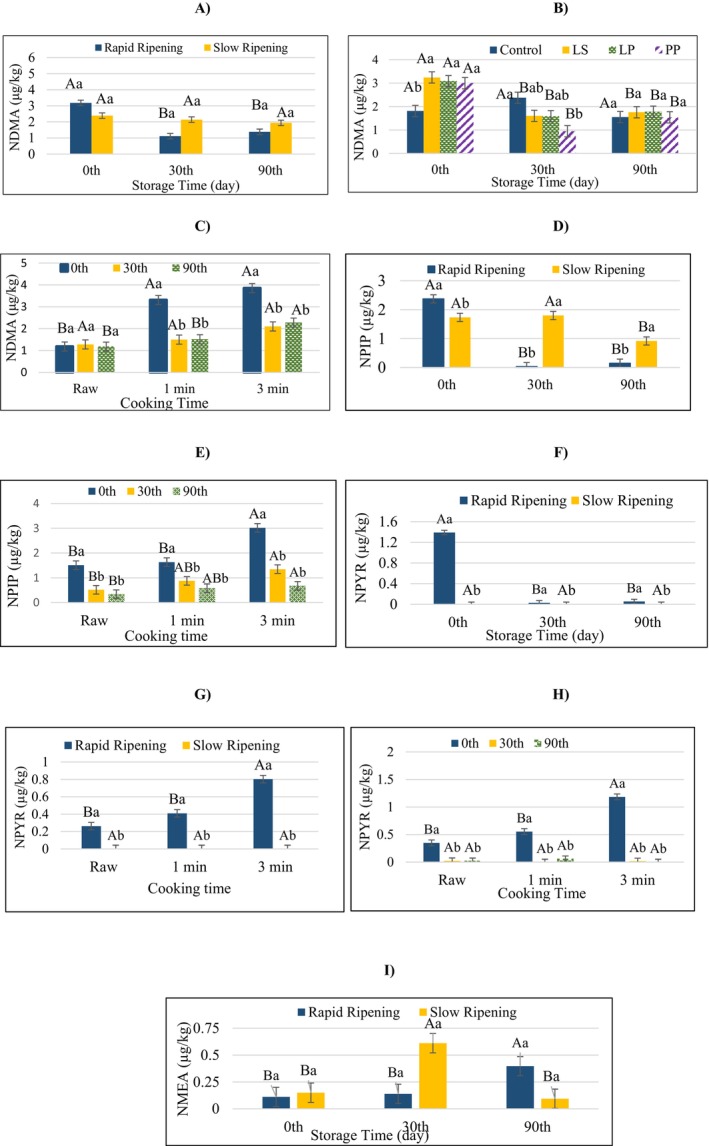
The effects of the interactions of the factors (ripening rate, storage time, starter culture, or cooking time) for NDMA (A–C), NPIP (D, E), NPYR (F–H), or NMEA (I). a and b: Different lowercase letters indicate significant differences between samples produced at the different ripening rates for the storage time. A and B: Different capital case letters indicate significant differences between samples during the storage time for the ripening rate (for A, D, F, I). a and b: Different lowercase letters indicate significant differences between samples produced with or without starter culture for the storage time. A and B: Different capital case letters indicate significant differences between samples during the storage time for with or without starter culture (for B). a and b: Different lowercase letters indicate significant differences during the storage time for the cooking degree. A and B: Different capital case letters indicate significant differences between samples cooked with different degrees or not for storage time (for C, E, H). a and b: Different lowercase letters indicate significant differences between samples produced at the different ripening rates for the cooking degree. A and B: Different capital case letters indicate significant differences between samples cooked with different degrees or not for ripening rate (for G) (*p* < 0.05).

The overall effect of ripening rate, starter culture, storage time, and cooking time on the nitrosamine levels of sucuk was given in Table 1. NDMA, classified as Group 2A by IARC, is a volatile nitrosamine that is widely determined in fermented sausages (Kızılkaya et al. 2023). Ripening rate and starter culture were determined to have no significant effect on NDMA (*p* > 0.05). However, the interactions of ripening rate × storage time had a significant effect on NDMA (*p* < 0.01) (Table 2). As can be seen in Figure 3A, there was no change in NDMA content in slow ripening during storage, but in rapid ripening, NDMA levels decreased significantly after the 30th day. It was thought that there may be a decrease in precursors starting from the 30th day in rapid ripening. On the other hand, significant changes in NDMA were also observed during storage depending on the LAB strains used (Figure 3B). While there was no significant difference in NDMA in the control group during storage, in the presence of autochthonous starter culture (LS, LP or PP), NDMA decreased on the 30th day and no significant change was observed on the 90th day (Figure 3B). According to the results, autochthonous strains brought about a decrease in NDMA level within the first 30 days of storage. This conclusion is thought to be related to the adsorption or metabolism of autochthonous strains (Nowak et al. 2014). According to Kim, Kim, et al. (2017), the *L. plantarum* (KCTC 3104) strain significantly reduced the amount of NDMA level. The same researchers also stated that this might be attributed to either the direct degradation of NDMA or the indirect degradation of precursors such as nitrite and dimethylamine. Furthermore, Xiao et al. (2018) expressed that the *L. pentosus* R3 strain forms an environment that directly breaks down nitrosamines.

**TABLE 2:** Overall effects of ripening rate, starter culture, and storage time on nitrosamine levels of sucuk (mean ± SE).

Treatment	*n*	Nitrosamines (μg/kg)			
		NDMA	NPIP	NPYR	NMEA
Ripening Rate (RR)					
Rapid	36	1.89 ± 0.10a	0.85 ± 0.08b	0.49 ± 0.03a	0.22 ± 0.05a
Slow	36	2.16 ± 0.10a	1.48 ± 0.08a	7.633E‐17 ± 0.03b	0.29 ± 0.05a
Significance		ns	**	**	ns
Starter Culture (SC)					
Control	18	1.91 ± 0.14a	1.18 ± 0.12a	0.23 ± 0.04a	0.18 ± 0.07a
LS	18	2.20 ± 0.14a	1.21 ± 0.12a	0.23 ± 0.04a	0.31 ± 0.07a
LP	18	2.15 ± 0.14a	1.15 ± 0.12a	0.26 ± 0.04a	0.26 ± 0.07a
PP	18	1.83 ± 0.14a	1.14 ± 0.12a	0.26 ± 0.04a	0.26 ± 0.07a
Significance		ns	ns	ns	ns
Storage Time (day) (ST)					
0	24	2.78 ± 0.12a	2.05 ± 0.10a	0.69 ± 0.03a	0.13 ± 0.06b
30	24	1.63 ± 0.12b	0.92 ± 0.10b	0.01 ± 0.03b	0.38 ± 0.03a
90	24	1.66 ± 0.12b	0.53 ± 0.10c	0.03 ± 0.03b	0.25 ± 0.03ab
Significance		**	**	**	*
Cooking Time (CT)					
0 min	24	1.22 ± 0.12c	0.79 ± 0.10b	0.131 ± 0.03b	0.21 ± 0.06a
1 min	24	2.11 ± 0.12b	1.03 ± 0.10b	0.204 ± 0.03b	0.31 ± 0.06a
3 min	24	2.74 ± 0.12a	1.68 ± 0.10a	0.401 ± 0.03a	0.24 ± 0.06a
Significance		**	**	**	ns
*RR × SC*		ns	ns	ns	ns
*RR × ST*		**	**	**	**
*RR × CT*		ns	ns	**	ns
*SC × ST*		**	ns	ns	ns
*SC × CT*		ns	ns	ns	ns
*ST × CT*		**	**	**	ns

*Note:* a–b: means marked with different letters in the same column are statistically different for each factor (ripening rate, starter culture, storage time, and cooking time).

Abbreviations: LP, *Lactiplantibacillus plantarum* S91; LS, *Latilactobacillus sakei* S15; ns, not significant; PP, *Pediococcus pentosaeceus* S128b; SE, standard error.

* *p* < 0.05.

** *p* < 0.01.

NDMA was reported to increase in fermented sausage with increasing heat treatment intensity (Li et al. 2012; Sallan et al. 2019, 2020). In the present study, the interaction of cooking time × storage time had a significant effect on NDMA, while the interactions of cooking time with other factors were not found to be significant. As can be seen in Figure 3C, while at the beginning of storage, the NDMA content increased when cooked for 1 min, an increase was observed only after 3 min of cooking in the samples stored for 90 days (Figure 3C). On the other hand, storage had no effect on the NDMA content of sucuk in raw samples (Figure 3C). These results are thought to be related to residual nitrite levels in sucuk samples. Indeed, it has been previously determined that the amount of residual nitrite plays a crucial role in nitrosamine formation (Popelka 2016; Sallan et al. 2019, 2020).

#### 3.5.2 N‐Nitrosopiperidine (NPIP)

NPIP is another nitrosamine that is frequently detected in fermented sausages. Black pepper contains piperine and piperidine, which may serve as precursors to nitrosopiperidine (NPIP). It has also been widely used in sucuk production (Sallan et al. 2019). Starter culture factor and its interaction with other factors did not show a significant effect on NPIP (*p* > 0.05) (Table 2). However, as can be seen from Figure 3D, a decrease in NPIP content was detected after 30 days of storage in rapid‐ripened samples, while on the 90th day in slow‐ripened samples. In addition, slow‐ripened samples gave lower NPIP values than rapid‐ripened ones at the beginning of storage. These findings are assumed to be linked to pH and residual nitrite level. Indeed, the residual nitrite level decreased as the storage period progressed in both rapid ripening and slow ripening conditions (Figure 2F). In the present study, slow ripening showed differences in terms of pH, lipid oxidation, and residual nitrite compared to rapid ripening. These differences are thought to affect the formation of nitrosamines.

NPIP was not significantly affected by starter culture (*p* > 0.05). In contrast, NPIP level had been significantly influenced by ripening rate (*p* < 0.01). In addition, the ripening rate × storage time interaction had a very important effect on this nitrosamine (Table 2). The decrease in NPIP level was detected after the 90th day in slow ripening and after the 30th day in rapid ripening (Figure 3D). On the other hand, at the beginning of storage, NPIP showed a significant increase when cooked for 3 min. In samples stored for 30 days, NPIP content increased as the cooking time increased, but the sample cooked for 1 min did not show a significant increase compared to the raw sample. A similar result was observed in the samples stored for 90 days. However, at all cooking levels (raw, 1 or 3 min), the stored samples showed lower NPIP levels (Figure 3E). This result shows that there is a slight decrease in NPIP precursors during storage. Sallan et al. (2019) and Kızılkaya et al. (2023) also noted that the extent of cooking is an important factor for NPIP level in sucuk.

#### 3.5.3 N‐Nitrosopyrrolidine (NPYR)

Starter culture and its interactions with the other factors were not found to have an important effect on NPYR (*p* > 0.05) (Table 2). The storage time × ripening rate interaction in Figure 3F indicated that rapid ripening resulted in a higher NPYR value for the samples at the beginning of the storage. In slow ripening, the storage factor showed no significant effect on NPYR. As seen in Figure 3G, rapid ripened samples gave a higher NPYR in 3 min of cooking compared to raw and 1 min cooked samples, while cooking time did not have any effect in the slow ripened group. Rapid ripening gave a higher NPYR than slow ripening in all three cooking times. However, in raw, 1 min, or 3 min cooking processes, the highest mean NPYR level was determined in the samples at the beginning of the storage, whereas no significant effect of cooking was observed in stored samples (Figure 3H). NPYR level was reported to increase with cooking but it is not parallel to the degree of cooking (Yurchenko and Mölder 2007; Sallan et al. 2019). At temperatures higher than 175°C, proline decarboxylation converts NPYR into pyrrolidine. Proline and pyrrolidine are transformed into NPYR when heat treatment is applied to cured meats (De Mey et al. 2017). On the other hand, Sallan et al. (2020) stated that an extreme decrease in pH (pH: 4.78) promotes NPYR formation. Proline and pyrrolidine have also been determined to facilitate the synthesis of NPYR, with pyrrolidine being more effective than nitrite concentration in this respect (Drabik‐Markiewicz et al. 2010).

#### 3.5.4 N‐Nitrosomethylethylamine (NMEA)

NMEA was not significantly affected by starter culture or cooking time (*p* > 0.05). NMEA content increased on the 90th day in rapid ripening. In slow ripening, NMEA level increased at the 30th day and afterwards decreased again (Figure 3I). The processing conditions applied and/or different raw material sources were reported to promote the formation of certain nitrosamines (Herrmann, Duedahl‐Olesen, and Granby 2015). Additionally, previous studies had shown for NMEA that fermented sausages either included very little or none (Herrmann, Duedahl‐Olesen, and Granby 2015; Niklas et al. 2022).

## 4 Conclusion

In this study, strains used as starter culture showed good growth. *Micrococcus/Staphylococcus* was affected by the ripening rate and gave higher values in slow ripening. Both in rapid and slow ripening, LS and LP caused a significant decrease in pH values compared to PP. In rapid ripening, TBARS values increased as storage time progressed, while in slow ripening, an increase was observed only on the 90th day. Starter culture had no significant effect on residual nitrite in slow ripening. The L* and a* values were not significantly impacted by the ripening rate. In terms of NDMA, the control group showed no significant change during storage, whereas in the presence of autochthonous starter culture (LS, LP or PP), NDMA content decreased on the 30th day and showed no significant difference on the 90th day. Starter culture and its interaction with other factors were not found to have a significant effect on NPYR, NPIP, and NMEA. The highest NPIP values were detected at the beginning of storage in both raw and cooked samples. While NDMA content increased at the beginning of storage when cooked for 1 min, an increase was observed only after 3 min of cooking in samples stored for the 90th day. Consequently, the decrease in NDMA level in the presence of autochthonous strains is thought to be related to the adsorption and metabolism of the strains. It is also assumed that this result is related to the degradation of precursors during storage. In addition, cooking the sucuk, especially for up to 3 min, increased the risk of nitrosamine in this product.

## Author Contributions

**Selen Sallan:** formal analysis (equal), investigation (equal), methodology (equal), project administration (lead), writing – original draft (lead), writing – review and editing (equal). **Zeynep Feyza Yılmaz Oral:** formal analysis (equal), investigation (supporting), methodology (equal). **Yağmur Akyol:** formal analysis (equal). **Güzin Kaban:** data curation (equal), methodology (equal), validation (lead), writing – review and editing (supporting). **Mükerrem Kaya:** data curation (equal), funding acquisition (equal), methodology (equal), supervision (lead), writing – review and editing (equal).

## Ethics Statement

The authors declare that they have no known competing financial interests or personal relationships that could have seemed to affect the work presented in this paper. No human or animal subjects participated in the experiment. All participants provided informed consent.

## Conflicts of Interest

The authors declare no conflicts of interest.

## Supporting information


**TABLE S1:** Linear range, limit of detection, limit of quantification, linear equality, correlation coefficient, recovery and relative standard deviation values of nitrosamines.

## Data Availability

Research data are not shared.
