# Supplementary material for: Evaluating the Role of Autochthonous Lactic Acid Bacteria and Ripening Conditions on Nitrosamine Inhibition in Sucuk Over Storage Time
Source: Food Sci Nutr. 2025 Aug 25;13(9):e70851. doi: 10.1002/fsn3.70851 (PMC12376181; doi:10.1002/fsn3.70851)
Supplement: Supplementary file 1 — TABLE S1: Linear range, limit of detection, limit of quantification, linear equality, correlation coefficient, recovery and relative standard deviation values of nitrosamines. [file FSN3-13-e70851-s001.docx]

**TABLE S1** Linear Range, Limit of Detection, Limit of Quantification, Linear Equality, Correlation Coefficient, Recovery and Relative Standard Deviation values ​​of nitrosamines

|  | Linear range (μg/mL) | Limit of Detection LOD) (μg/mL) | Limit of Quantification (LOQ) (μg/mL) | Linear equality | Correlation Coefficient (R^2^) | Recovery (%) | Relative Standard Deviation (RSD%) |
| --- | --- | --- | --- | --- | --- | --- | --- |
| NDMA | 0.5-20 | 0.32 | 0.98 | y=39157x-9242.9 | 0.9999 | 101-104.37 | 4.44-6.15 |
| NMEA | 0.5-20 | 0.42 | 1.28 | y=37559x-524.68 | 0.9999 | 97-101.07 | 1.85-6.78 |
| NDEA | 0.5-20 | 0.44 | 1.34 | y=37649-637.88 | 0.9999 | 94-101.36 | 3.06-7.42 |
| NPYR | 0.5-20 | 0.36 | 1.09 | y=28751x-2272.5 | 0.9999 | 95.43-103.17 | 2.98-7.12 |
| NPIP | 0.5-20 | 0.32 | 0.98 | y=32293x-7776.8 | 0.9999 | 99.73-100.83 | 0.95-4-56 |
| NDBA | 0.5-20 | 0.38 | 1.15 | y=13616x-3859.9 | 0.9999 | 96.97-99.79 | 1.26-5.81 |
| NDPA | 0.5-20 | 0.15 | 0.46 | y=6376.7x-3529.3 | 0.9999 | 97.17-99.79 | 0.77-5.77 |
